# Nebivolol Ameliorates Nitric Oxide–Deficient Hypertension

**DOI:** 10.1100/tsw.2002.814

**Published:** 2002-06-20

**Authors:** L.A. Fortepiani, M.C. Ortiz, N.M. Atucha, Joaquin Garcia-Estan

**Affiliations:** Departamento de Fisiología, Facultad de Medicina, 30100 Murcia, Spain

**Keywords:** arterial hypertension, renal function, beta adrenergic blockers, nitric oxide, plasma renin activity

## Abstract

Nebivolol is a new selective beta 1-adrenoceptor antagonist with nitric oxide (NO)–releasing properties. In the present study we have analyzed whether nebivolol affects the development of the arterial hypertension that follows the chronic inhibition of nitric oxide synthesis. Nebivolol (1 mg/kg/day, 14 days) was given concurrently with the NO synthesis inhibitor N-nitro-L-arginine methyl ester (L-NAME, 0.1, 1, and 10 mg/kg/day, 14 days) to several groups of rats. Blood pressure, renal function, plasma renin activity (PRA), and NO activity and metabolites were measured at the end of the treatment period. L-NAME treatment alone increased mean arterial pressure dose dependently (103.5 ± 2.4, 110.9 ± 2.0, and 125.8 ± 2.2 mmHg, respectively). Nebivolol completely prevented the development of arterial hypertension in the groups treated with L-NAME at the doses of 0.1 and 1 mg/kg/day and reduced the increase achieved with the L-NAME dose of 10 mg/kg/day (110.3 ± 2.7). There were no differences in glomerular filtration rate or natriuresis between nebivolol-treated and -untreated rats. Plasma nitrates+nitrites and calcium-dependent NO synthase activity in the kidney also decreased dose dependently with L-NAME treatment and nebivolol did not significantly modify it. However, PRA was lower in all groups treated with nebivolol and L-NAME as compared to the rats receiving only L-NAME. These data indicate that nebivolol prevents the development of the arterial hypertension associated with chronic NO deficit and this effect seems to be dependent on the inhibition of renin-angiotensin system.

### INTRODUCTION

It is now well known that chronic inhibition of NO production produces arterial hypertension[1,2,3,4,5]. The mechanisms underlying this effect are not completely established but there is evidence suggesting that the renin-angiotensin system is in big part responsible for the renal and systemic alterations induced by the chronically reduced NO availability. Thus, chronic angiotensin II blockade or converting enzyme inhibition prevents the development of $N^w$-nitro-L-arginine methyl ester (L-NAME)–induced hypertension[6,7,8,9,10,11]. However, it seems that these treatments cannot overcome the effect of L-NAME and therefore stimulate the reduced synthesis of NO[12,13], which suggests that these treatments cannot completely revert the altered physiological status of the L-NAME–treated arterial hypertension model, that is to normalize NO synthesis.

Among several new drugs already available that can fulfill the requirement of increasing NO synthesis, a new class is especially interesting. Beta 1-adrenoceptor antagonists with NO-releasing properties, such as nebivolol[14,15], could be important tools to improve the arterial hypertension and associated abnormalities secondary to the chronic deficiency of NO, because of two main effects. One is the above-mentioned increase in NO production; the other is the plasma renin activity (PRA)–suppressing effect. Therefore, in the present study we have evaluated the effect of nebivolol in a model of chronic deficiency of NO and arterial hypertension.

### METHODS

Male Sprague-Dawley rats (Harlan, Barcelona, Spain) were used for the study. The animals were maintained on standard rat chow and tap water throughout the study. All experiments were performed according to the guidelines for the ethical treatment of the animals of the European Union.

#### Experimental Groups

The animals (around 250 g) were randomly assigned to one of eight groups (n = 8 each, except where noted). The first three groups received L-NAME (Fluka, Madrid, Spain) at the doses of 0.1, 1, and 10 mg/kg/day, respectively. Three more groups received these same doses and also concurrently nebivolol (1 mg/kg/day). Another group (n = 6) received only nebivolol at the same dose. Finally, a control untreated group was prepared. All the treatments were given in the drinking water for 15 days. The optimum dosage of the drugs was adjusted daily according to the water intake and body weight of the animals. L-NAME was dissolved in water and nebivolol in 5% glucose.

#### Experimental Protocol

Blood pressure was determined by the tail cuff method (Cibertec, Madrid, Spain) by using MacLab software (AD Instruments, U.K.) in a Macintosh LCII computer. This approach allowed the estimation of systolic and diastolic blood pressures, and then of mean arterial pressure, following the analysis of pulse signal, which also permitted the calculation of heart rate. In previous experiments, we observed that the precision of this procedure was more than 95% as compared to the direct intra-arterial recording[4]. The measurements were made the day before starting the treatments, and 7 and 14 days later. After this last blood pressure measurement, the animals were housed in metabolic cages for 24 h to obtain urine, during which time they drank their usual drug combination. Then, at the end of this period, the animals were anesthetized with Inactin (100 mg/kg, i.p., Research Biomedical International, Natick, MA) and blood was obtained from cardiac puncture and uncoagulated with 6% EDTA. After killing the animals with anesthetic overdose, both kidneys were taken out and immediately frozen at -80ºC to measure NO synthase activity. After centrifugation at 4ºC at 2,500*g*, plasma was separated and frozen at –20ºC to measure PRA, plasma nitrites+nitrates, creatinine, and sodium. Urine samples were measured by gravimetry, centrifuged at the same speed, and frozen also to measure creatinine and sodium.

### Analytical Techniques

Glomerular filtration rate (GFR) was estimated as the clearance of creatinine (urine to plasma concentration ratio times diuresis), and was normalized per 100 g of body weight. Plasma and urine creatinine were measured by the Jaffe reaction using a commercially available kit (Boehringer Manheim, Barcelona, Spain). Sodium concentration was measured by flame photometry (Corning 435, Izasa, Barcelona, Spain). PRA was measured by a commercially available kit (DiaSorin, Saluggia, Italy). Calcium-dependent NO synthase activity was measured by the conversion of [3H]-arginine to [3H]-citrulline in kidney homogenates as previously described[16]. Plasma nitrates+nitrites were measured by capillary electrophoresis following a method previously described[17].

### Statistical Methods

Data are presented as means ± S.E.M. A repeated measures analysis of variance was used to obtain the statistical significance between and within groups. If the global analysis was significant, a post hoc Duncan's test was carried out. Differences were considered statistically significant at a *p* level lower than 0.05.

## RESULTS

The dose of nebivolol used in the present experiments was selected according to the literature[14,15] and based on preliminary experiments which showed that 1 mg/kg/day of nebivolol for 14 days reduced basal heart rate in a group of normal anesthetized rats (control: 438.0 ± 2.6 beats/min; nebivolol-treated: 343.3 ± 6.9, n = 4 each).

Treatment with L-NAME induced a progressive and dose-dependent elevation in mean arterial pressure (MAP), as shown in Fig. 1 (left panel). Thus, MAP increased 7.6 ± 2.8 mmHg with the dose of 0.1 mg/kg/day, 12.6 ± 3.9 with the dose of 1 mg/kg/day and 23.5 ± 3.3 with the dose of 10 mg/kg/day. Nebivolol (Fig. 1, right panel) completely prevented the elevation of MAP in the groups treated with 0.1 and 1 mg/kg/day of L-NAME (a change of –2.5 ± 1.8 and –3.2 ± 1.7 mmHg, respectively) and significantly reduced the final MAP levels in the animals treated with 10 mg/kg/day of L-NAME (an increase of 11.2 ± 2.4 mmHg). In the control group, MAP was well maintained throughout the study period and averaged 104.3 ±1.5 at the end of the study. The MAP of the animals chronically treated with nebivolol only was 97.5 ± 2.6.

Heart rate did not change significantly with L-NAME treatment, but in the groups treated also with nebivolol it was lower at the end of the treatment (Fig. 2).

There were no important changes in renal function (Fig. 3). GFR in the control group was 1.5 ± 0.1 ml/min/g and L-NAME treatment slightly decreased these values, but without differences between them. Nebivolol treatment had a tendency to improve GFR, but only the nebivolol-treated group receiving 10 mg/kg/day reached higher values than its control (Fig. 3, left panel). The excretion of sodium was similar between nebivolol-treated and -untreated groups (Fig. 3, right panel).

PRA was 3.5 ± 0.4 ng/ml/h in the control group. L-NAME treatment increased PRA in all three L-NAME–treated groups but was higher in the group receiving the lower L-NAME dose (Fig. 4). Nebivolol significantly decreased PRA in all three groups and these values were not significantly different to the control group.

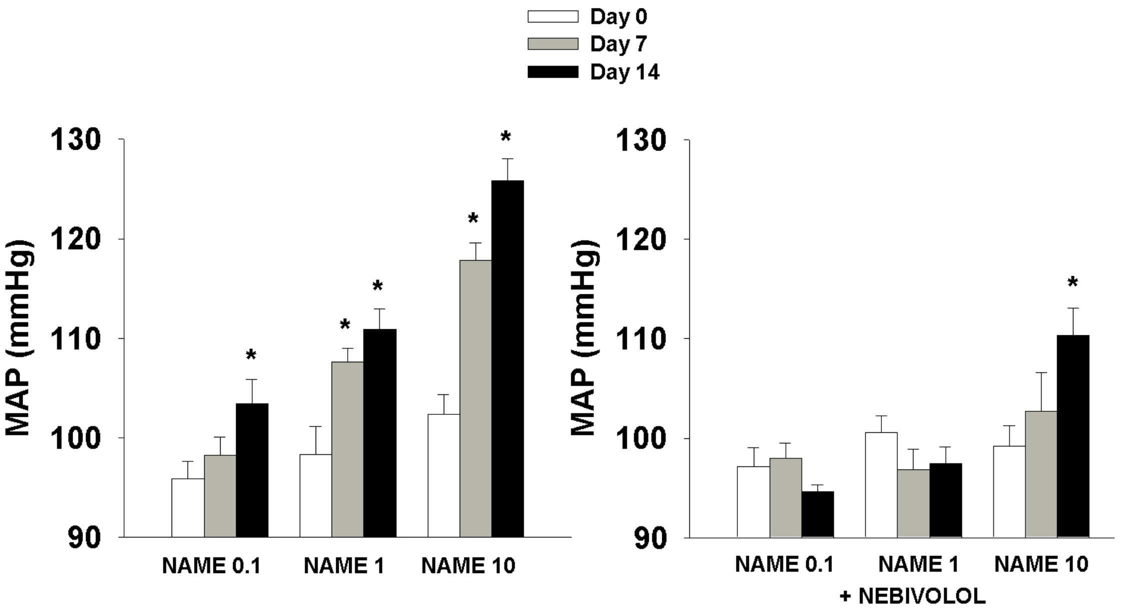

**FIGURE 1**. Time-dependent changes in mean arterial pressure (MAP) in the experimental groups. *, $p < 0.05$ vs. day 0.

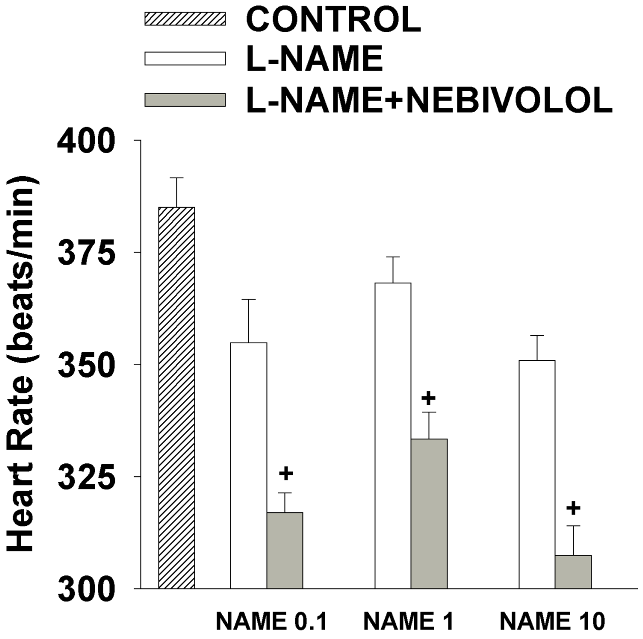

**FIGURE 2**. Heart rate in the experimental groups after 2 weeks of treatment. +, $p < 0.05$ vs. L-NAME–treated group.

Calcium-dependent NO synthase activity measured in kidney homogenates was lower in the L-NAME–treated groups than in the control (30.2 ± 4.6 pg/min/g). L-NAME treatment also reduced NO synthase activity dose dependently. Nebivolol did not increase significantly NO synthase activity in any L-NAME–treated group (Fig. 5, left panel).

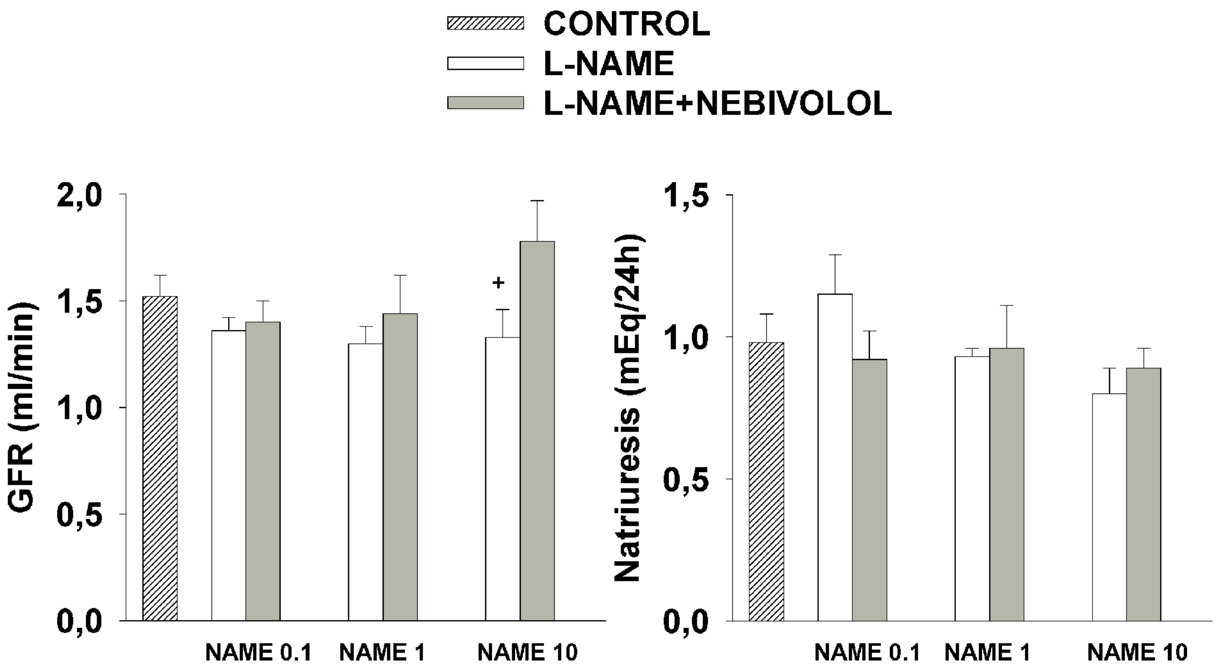

**FIGURE 3**. Glomerular filtration rate (left) and sodium excretion (right) after 2 weeks of treatment. +, $p < 0.05$ vs. L-NAME–treated group.

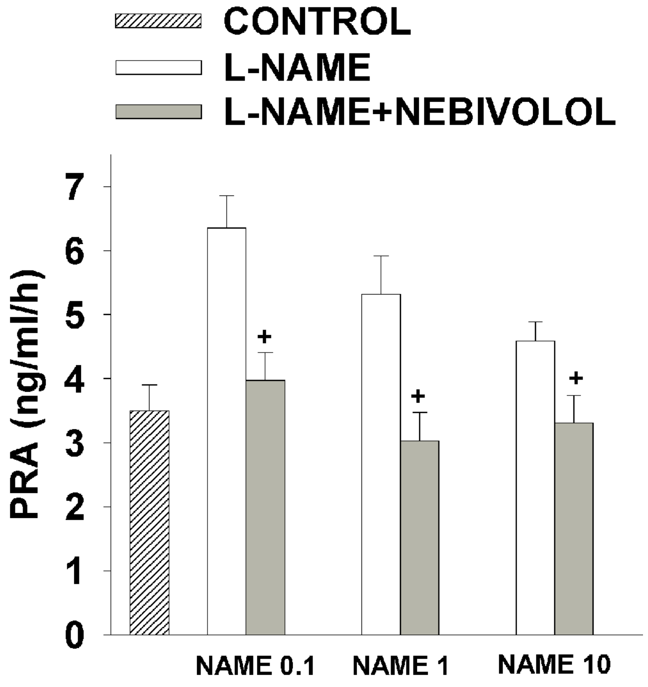

**FIGURE 4**. Plasma renin activity (PRA) in the experimental groups after 2 weeks of treatment. +, $p < 0.05$ vs. L-NAME–treated group.

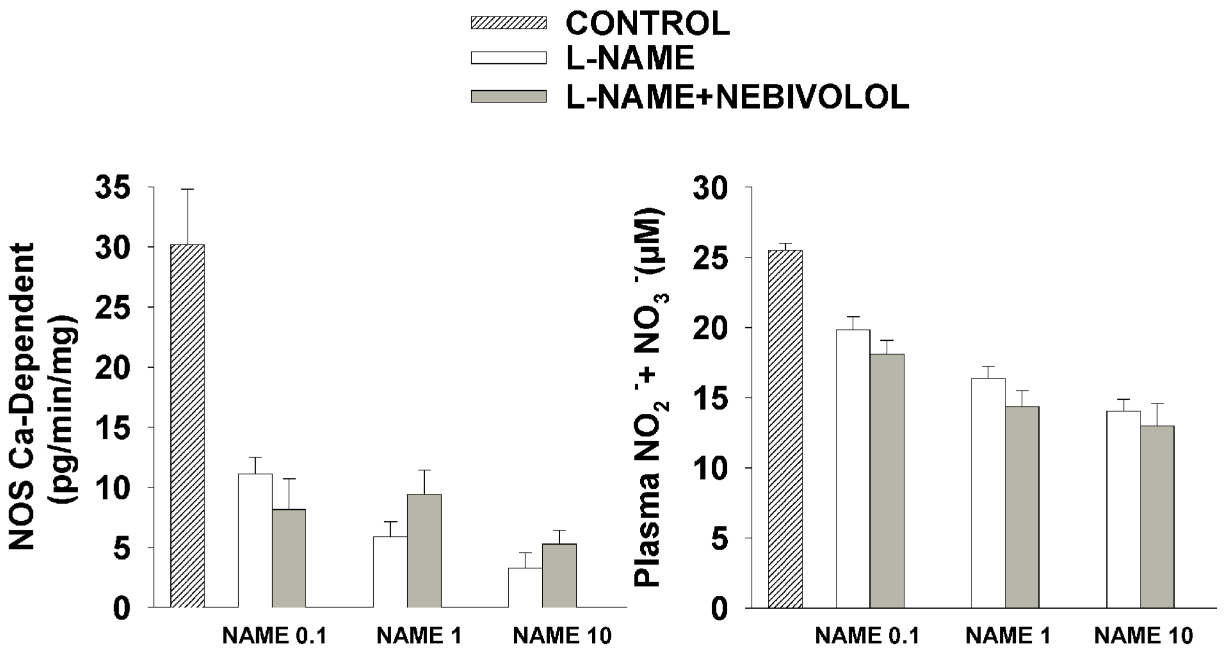

**FIGURE 5**. Renal calcium-dependent nitric oxide synthase (NOS) activity (left) and plasma nitrates+nitrites (right) in the experimental groups after 2 weeks of treatment.

Also, plasma nitrates+nitrites was significantly lower in the L-NAME–treated groups than in the control (25.5 ± 0.5 µM) and nebivolol treatment did not changes these lower values (Fig. 5, right panel).

### DISCUSSION

In the present study we have analyzed the effects of a new selective beta 1-adrenoceptor antagonist, nebivolol, to improve the arterial hypertension associated with the chronic deficiency of nitric oxide (NO). The results indicate that nebivolol is an effective treatment for the arterial hypertension that follows the chronic inhibition of NO.

Previous studies have shown that chronic L-NAME administration produced a dose-dependent form of arterial hypertension[3,4] and the present results agree with these data, since administration of three doses of the NO synthesis inhibitor increased MAP in a dose-dependent form. Most investigators agree in that this elevation of blood pressure is dependent on an increased activity of the renin-angiotensin system[6,7,8,9,10,11]. Thus, NO is a potent inhibitor of renin release and its inhibition should elevate renin production. In fact, previous studies utilizing high doses of NO synthesis inhibitors were unable to document clearly elevated renin levels[3,11,19]. This fact was difficult to reconcile with other studies showing that converting enzyme inhibition or angiotensin receptor antagonism prevented the development of this type of arterial hypertension. However, it seems that low doses of the NO inhibitors in fact do elevate renin levels, whereas high doses tend to suppress it probably because of the elevated blood pressure levels and also as a consequence of cortical renal ischemia[20]. The present results completely agree with this view and show that the lower doses of L-NAME produce greater PRA levels and they decrease as the L-NAME doses increase. Thus, it is interesting to see that the MAP levels are higher in the group with lower PRA values and this is probably due also to the lower production of NO.

Nebivolol treatment concurrently with L-NAME was clearly beneficial for these animals, since the arterial hypertension elicited by the lower two doses of the inhibitor was completely prevented and reduced 50% in the case of the group receiving the higher L-NAME dose. Also, heart rate was lower in the animals treated with nebivolol, suggestive of a cardiac effect of nebivolol. Among the possible mechanisms by which nebivolol can exert this antihypertensive effect, we have analyzed two. First, functioning as a beta-adrenergic blocker, PRA should be inhibited and in fact it was. All the three groups of animals treated with nebivolol showed lower PRA values than their respective hypertensive groups. Second, nebivolol has been shown to be able to release NO[14,15,18]. Thus, as an index of NO production, we measured NOS activity in the kidney, an organ that is thought to be very sensitive to the effect of NO inhibition[21]. However, calcium-dependent NOS activity in kidney homogenates, which was dose dependently reduced by L-NAME treatment, was not significantly modified in the groups that received nebivolol. However, a possible elevation of NO production in peripheral vascular beds other than the kidney, locally relevant and contributing to the observed MAP value reduction, but not sufficient to sensibly modify circulating nitrites-nitrates levels, can not be ruled out. Similarly, the lower plasma nitrates+nitrites levels of the L-NAME–treated rats were not changed by nebivolol. Thus, these results therefore suggests that the hypertensive effect brought about by L-NAME treatment is abrogated or reduced by the inhibition of renin production and not by an enhanced production of NO.

Previous studies have shown that nebivolol produces relaxation of vascular beds[14,15] and releases NO from the kidney[18], effects that are subsequently blocked by NO synthesis inhibitors. In the present study, the group of animals treated chronically with nebivolol showed a lower blood pressure than those simultaneously treated with nebivolol and L-NAME, which suggests that this difference in blood pressure may be due to the NO releasing properties of nebivolol. However, the present results also suggest that nebivolol is not able to overcome a simultaneous competitive NOS inhibition, such as that produced by L-NAME. Therefore, it is possible that the mechanism by which nebivolol releases NO is not based on an activation of the NOS enzyme. However, other possibilities cannot be ruled out, such as a central nervous system effect or a presynaptic inhibition that would decrease norepinephrine release from sympathetic nerve endings. It is important, however, to keep in mind that we have used a very low dose of nebivolol, and it is possible that a higher dose might be needed in order to fully counteract the NOS inhibition. Then, the elucidation of the exact mechanism by which nebivolol increases NO production will require further studies.

Regarding the renal function data, the present results agree with previous data from this and other laboratories[3,5,22,23] showing a decrease in the GFR and a maintenance of the sodium excretion levels. Since blood pressure was elevated, the resulting pressure-natriuresis relationship is reduced, as we have previously demonstrated[5,23]. Nebivolol did not importantly alter these values, except in the group receiving the highest L-NAME dose, although clearly the pressure natriuresis curves were normalized in the groups receiving the lowest two doses of L-NAME and improved in the group receiving the highest L-NAME dose.

In summary, nebivolol prevents the development of the arterial hypertension induced by chronic inhibition of NO synthesis. This effect seems to be mediated by a PRA-suppressing effect and not by an enhancement of NO production. Overall, these data indicate that nebivolol is a beneficial drug in situations of chronically reduced NO availability. Therefore, this study suggests that, besides converting enzyme inhibitors and angiotensin receptor antagonists, nebivolol, at least in part through its beta-blocking activity, is a new therapeutic option to treat conditions originated by chronically reduced NO availability.

### ACKNOWLEDGMENTS

This study has been supported by a research grant from Laboratorios Menarini (Barcelona, Spain). They also provided us with nebivolol hydrochloride ((+)-[(SRRR)+(RSSS)]-α,α′-[iminobis

(methylene)]*bis*[6-fluoro-3,4-dihydro-2*H*-1-benzopyran-2-methanol] hydrochloride). The authors thank to Dr. María Dolores Alcazar and Magdalena, of the Servicio de Instrumentación Científica of the Universidad de Murcia, for her expert work on the measurements of plasma nitrates+nitrites.

### BIOSKETCHES

**Joaquin Garcia-Estan**, M.D., Ph.D., is Professor of Physiology, University of Murcia (Spain). His research interests include analysis of mechanisms of diseases such as arterial hypertension and liver cirrhosis from the renal and vascular points of view.

**Lourdes A. Fortepiani**, M.D., Ph.D., is postdoctoral fellow, University of Murcia (Spain). Her research interests include analysis of mechanisms of disease in arterial hypertension from the renal and vascular points of view.

**M. Clara Ortiz**, M.D., Ph.D., is postdoctoral fellow, University of Murcia (Spain). Her research interests include analysis of mechanisms of disease in liver cirrhosis from the renal and vascular points of view.

**Noemí M. Atucha**, M.D., Ph.D., is Professor of Physiology, University of Murcia (Spain). Her research interests include analysis of mechanisms of disease in arterial hypertension from the renal and vascular points of view.
